# Analysis of the lymphocyte cell population during malaria caused by *Plasmodium vivax* and its correlation with parasitaemia and thrombocytopaenia

**DOI:** 10.1186/s12936-018-2443-x

**Published:** 2018-08-20

**Authors:** Samantha Soares Ourives, Quessi Irias Borges, Diego Sampaio Arantes dos Santos, Eponina Cláudia Magalhães Melo, Rodrigo Medeiros de Souza, Amílcar Sabino Damazo

**Affiliations:** 1 Faculty of Medicine (FM), Federal University of Mato Grosso (UFMT), Cuiabá, Mato Grosso 78060-900 Brazil; 2 Department of Basic Science in Health Faculty of Medicine (FM), Federal University of Mato Grosso (UFMT), Cuiabá, Mato Grosso 78060-900 Brazil; 3 Centre for Health Sciences and Sport, Federal University of Acre (UFAC), Cruzeiro do Sul, AC 69980000 Brazil; 4 Hospital of the Woman and the Child of the Juruá-Laboratory of Clinical Analysis, Cruzeiro do Sul, AC 69980000 Brazil

**Keywords:** *Plasmodium vivax*, Parasitaemia, Platelets, Th1 cells, Th2 cells

## Abstract

**Background:**

The mechanisms of activation and regulation of T lymphocytes and their cytokines in malaria caused by *Plasmodium vivax* are complex and poorly understood. Previous data suggest that T cells balance protective immune responses with immune mediated pathology in malaria. This study investigates the lymphocytic profile of patients infected with *P. vivax* by identifying and quantifying the specific sub-populations of Th1, Th2, Th17 and Treg cells and observing the correlation between parasitaemia and the number of platelets.

**Methods:**

A cross-sectional study was carried out in an endemic area of the state of Acre, Brazil. In order to obtain identification and quantification of lymphocyte sub-populations through flow cytometry, blood samples were collected from 50 individuals infected with *P. vivax* and 20 non-infected controls. To differentiate Th1 from Th2, the presence of cytokines IL-4 and TNF was examined by enzyme-linked immunosorbent assay. Utilizing the Mann–Whitney and Spearman coefficient tests, comparison and correlation analysis were rendered to test the parasitaemia and the number of platelets relationship.

**Results:**

The data indicate that individuals infected with *P. vivax* present a significant reduction in Th1, Th2 and Th17 cell sub-populations when compared to the non-infected control group. A negative correlation exists between parasitaemia and platelet counts in individuals infected with *P. vivax*. There is no correlation of parasitaemia or thrombocytopaenia with any sub-population of T lymphocytes analysed. Interestingly, patients with serum Th1 cytokine profile present inversely proportional parasitaemia to the increase in the number of Th1, Th2, Th17 and Treg cells while patients with serum Th2 cytokine profile present directly proportional parasitaemia to the increase in number of Th1 and Th2 cells. Regarding the number of platelets, patients with serum Th1 cytokine profile show a correlation directly proportional to the Th17 sub-population. In contrast, platelet counts are directly proportional only to Treg and activated Treg cells in patients with serum Th2 cytokine profile.

**Conclusions:**

During the *P. vivax* infection patients with serum Th1 versus Th2 cytokine profile present different biological mechanisms for activating the immune system against parasite load.

**Electronic supplementary material:**

The online version of this article (10.1186/s12936-018-2443-x) contains supplementary material, which is available to authorized users.

## Background

*Plasmodium vivax* is one of five parasites causing malaria in humans. There has been an increasing amount of documentation referring to the disease as a major health threat affecting the world’s most populous regions [1]. During the infection, patients present leukogram variations with values ranging from normal to leukopaenia [2]. Moreover, *P. vivax* has a greater capacity to elicit an inflammatory response, resulting in a lower pyrogenic threshold, and activation and dysfunction of T cells [3]. *Plasmodium vivax* blood-stage infection activates a substantially different type of immune response compared to *Plasmodium falciparum* and might have distinct contributions to the immune response to blood-stage infection [4].

Anaemia is a constant finding in malaria and progresses with disease proliferation [5]. Another finding frequently observed in *P. vivax* infection is thrombocytopaenia. Several hypotheses have already been postulated as causes of malaria-associated thrombocytopaenia, including but not limited to: disseminated intravascular coagulation, immune mechanisms, splenic sequestration, and the possible presence of the parasite in red blood cells in the bone marrow, with may lead to a decrease in the platelet population in circulation [6].

The pro‐inflammatory response against *P. vivax* gains more importance during periods of increased parasite burden [7]. Malaria parasites regulate the expression of selective Toll-like receptors (TLRs) on immune cells that induce a specific biological response against invasion of malaria parasites [8].

In malaria there is activation of both Th1 and Th2 cells. A balance between the cytokines produced by both cell profiles is required for the protection of the individual [9]. Elevated levels in Th1 and Th2 cytokines such as IFN-γ and IL-4 are associated with increased severity in some diseases [10]. Many individuals with asymptomatic malaria display multiple significant interactions involving IL-4 [11]. Studies have shown the protective role of the IL-4 cytokine as a negative regulator of the pro-inflammatory effects in malaria infection [11, 12]. IFN-γ is a cytokine of Th1 cells and plays an essential role in immunity against blood-stage *Plasmodium* infection [13]. Combined with TNF and CCL5 chemokine, they are proven to be crucial biomarkers in the profile of individuals with mild infection of *P. vivax*, and in contrast, there are positive correlations involving TNF and IFN-γ associated with pathogenicity in severe malaria [11].

Bueno and collaborators [14] have suggested the existence of different sub-populations of Treg cells during malaria infection, evidenced by the intracellular production of IFN-γ, IL-4 and IL-17 and the intermediate expression of the FOXP3 molecule. It should be emphasized, however, that not all FOXP3^+^ T cells are necessarily Treg cells and their activity may depend on the level of FOXP3 expression and isoforms of the protein expressed [15]. Some studies discuss the cellular and molecular factors that affect the development, the homeostasis and the function of the Treg cells and consequent immunity to self and non-self antigens [16]. The identification of CD127 receptor down-regulated in all human T cells after activation as a useful marker was heralded by genetic observations and a combination of CD4, CD25, and CD127 resulted in a highly purified population of Treg cells accounting for significantly more cells that previously identified based on other cell surface markers. These cells were highly suppressive in functional suppressor assays [15]. It is believed that Treg cells act to suppress the inflammatory response by obtaining direct contact with effector cells and via the production of cytokines such as IL-10 and TGF-β [17]. Also, Treg cells have been known to significantly modify cellular immune responses to various protozoan infections, including malaria [18]. However, these CD4^+^CD25^+^CD127^−^, once isolated, may be treated in vitro with TGF-β or other factors to enhance Treg cell function in these cells [15].

Another cell population recently associated with parasitic infections is Th17 cells. These cells are mediated by cytokines IL-17A and IL-17F, inducing the expression of pro-inflammatory cytokines as a consequence of the recruitment and activation of several leukocyte lines [19]. There are a few studies that described the importance of Th17 in the infection caused by malaria [20, 21]. Ishida and collaborators [21] demonstrated that the absence of IL-17 in transgenic animals favoured the development of cerebral malaria during *Plasmodium berghei* (ANKA strain) infection and point out the protective function of this cell in *P. berghei* ANKA malaria. Elevated IL-17 levels combined with high IL-4, IL-12α and IFN-γ levels may be a marker of protection. The mechanism may be controlled by host factor(s) [20].

Based on the literature, the aim of this study is to investigate the immunological profile of patients with acute malaria caused by *P. vivax.* The study analysed the association of platelets and the level of parasitaemia with the amount of sub-populations of lymphocytes: Th1, Th2, Th17 and Treg cells. The study also investigated haematological and biochemical parameters in patients with acute malaria caused by *P. vivax*, using haematological and biochemical markers. The final step compared the patients with *P. vivax* malaria to the non-infected control subjects.

## Methods

### Area of study

This is a cross-sectional descriptive study of patients in the acute phase of malaria caused by *P. vivax*, from September to December of 2016. Patients are located in the city of Cruzeiro do Sul, considered the second largest city in the state of Acre, Brazil.

### Patients

The patients (n = 50) diagnosed with vivax malaria were analysed in the acute phase of the disease before receiving immunosuppressive or anti-malarial drugs and were between 18 and 83 years of age, both sexes, with no other acute or chronic infections or pregnancy.

The diagnosis of *P. vivax* infection was confirmed microscopically by a thick gauge stained with 5% Giemsa. Individuals with a positive diagnosis who agreed to participate in the study after reading and signing the informed consent form were included. The age, number of previous episodes of malaria, and the history of other infectious diseases of each participant were recorded in a standard questionnaire during their care. In addition, a peripheral blood sample (15 mL) was collected for laboratory tests and analyses. Patients were informed that whether or not they participated in the project, it would not affect their attendance at the health centres in the city of Cruzeiro do Sul-Acre.

### Non-infected controls

In this study 20 healthy clinicians were selected for blood donation at the Haematology and Haemotherapy Centre of Mato Grosso-Brazil. Some of the requirements were no previous history of malaria and the clinicians had to be residents of regions considered non-endemic to the disease. Consent was obtained from all of the individuals evaluated for participation in the study. Haematological data were obtained for the comparison analyses. The exclusion criteria were the same as those used in the group of patients infected with *P. vivax*.

### Parasitological diagnosis

Thick smears were performed on 1-cm^2^ of slide with 1 drop of peripheral blood. This slide was stained with 5% Giemsa solution and examined to determine the *Plasmodium* species by two trained microscopists from light microscopic images (Axio imager Z2, Carl Zeiss).

The diagnosis of the species and the quantification of parasitaemia were carried out by counting the number of parasites in fields of high magnification (1,000×), per 200 leukocytes. If 9 or fewer parasites were found, 300 leukocytes were counted in addition. The estimation of parasitaemia per mm^3^ of blood was expressed as the relation between parasites and leukocytes in the blood smears. The patients were classified according to the level of parasitaemia (low up to 750 parasites/mm^3^ and high above 751 parasites/mm^3^) [22, 23].

### Laboratory analysis

The haemogram was performed on the same day of blood collection, from 3 mL of venous blood, collected in a Vacutainer^®^ tube with anticoagulant EDTA. Erythrocytes (millions/mm^3^), hemoglobin (g/dL), haematocrit (%), platelets (mm^3^) and total/differential leukocytes (%), were evaluated by automatic counting of blood cells (ABX Pentra 90, Horiba Diagnostics, Kyoto, Japan).

To make a biochemical analysis, a sample of 5 mL of venous blood was collected in a tube Vacutainer^®^ with separator gel to obtain the serum. After the clot formation, the serum was removed and the biochemical tests were immediately dosed, using specific Labtest kits of the following parameters: bilirubin, UV urea liquiform, creatinine, alanine amino transaminase (ALT/GPT) liquiform and aspartate amino transaminase (AST/GOT) liquiform in automatic analyzer Labmax 240 Premium (Labtest Diagnostica S/A, Brazil).

All of these procedures were performed at the Clinical Analysis Laboratory of the Juruá Women’s and Children’s Hospital in the city of Cruzeiro do Sul-AC by local professionals. The classification of severity of the patients analysed, followed the criteria for classification of severe falciparum malaria established by the World Health Organization (WHO). This model was chosen because there is not yet in the literature a specific gamut of severity for malaria caused by *P. vivax*.

### Cell immunophenotyping

For immunophenotyping analysis, venous blood (7 mL) was collected in tubes containing EDTA. Then, peripheral blood leukocytes isolation was obtained by centrifugation of whole blood. Then, the ring area of leukocytes was collected. Erythrocytes were lysed with a lyse kit (Sigma, USA). While cells were resuspended (1 × 10^7^ per 900 µL) in RPMI media with 20% of autologous serum and 10% DMSO (Sigma). Lastly, cell suspension was placed in cryotubes and frozen at − 80 °C [24] and transported to UFMT, Cuiabá-MT, by air transportation in appropriate temperature conditions and packaging.

For the immunophenotyping of T cell subpopulations: Th1 (CD3^+^CD4^+^IFN-γ^+^TNF-α^+^), Th2 (CD3^+^CD4^+^IL-4^+^), Th17 (CD3^+^CD4^+^IL-17^+^), Treg (CD4^+^CD25^+^CD127^+^FOXP3^+^) and activated Treg (CD4^+^CD25^+^CD127^−^FOXP3^+^), the blood samples (1 mL) were naturally thawed at room temperature and incubated for 5 min with 14 mL of lysis solution (NH_4_Cl/KHCO_3_/tetrasodium EDTA), centrifuged for 5 min at 300×*g*. The supernatant was aspirated and the pellet was resuspended in 5 mL of ice cold PBS and centrifuged for 5 min at 300×*g*. Again, the supernatant was aspirated and the pellet was resuspended in 1 mL ice-cold PBS solution [25].

Once cell lysis has taken place, the cells were resuspended in 100 μL of FACS solution (PBS + 0.5% BSA) containing specific combinations and concentrations of distinct fluorochromes labelled with monoclonal antibodies for simultaneous analysis of cell surface markers (Additional file 1: Table S1), and incubated for 30 min on ice and sheltered from light. After this period, 100 μL of Reagent A (Medium Fix & Perm, BD-Becton–Dickinson) solution was added and incubated for 15 min in the dark. Two 5-min washings at 350×*g* in FACS solution were performed and the cell contents resuspended in 100 μL of Reagent B (Permeabilization Medium-Fix & Perm, BD-Becton–Dickinson) solution and the specific volume in μL (Additional file 1: Table S1) for each intracellular labelled conjugated antibody was incubated for 20 min on ice and sheltered from light. Subsequently, the cells were centrifuged for 5 min at 350×*g* and resuspended in 200 μL FACS solution for immediate reading on the Accuri™C6 BD flow cytometer (Becton–Dickinson, USA), at the UFMT-Cuiabá Research Laboratory and the markers were performed according to the protocols proposed by the manufacturers (Additional file 1: Table S1).

Eighty thousand events were acquired and analysed by BD Accuri™ C6 software (BD Biosciences, USA). Isotype controls labeled with FITC, PE, PerCP, PerCP-CyTM 5.5 and APC were used in all experiments. The data obtained (percentage of fluorescent cells) were interpolated with the initial lymphocyte count obtained from the leukogram. The results are expressed as cells/mm^3^.

### Determination of patient immunological type

Patients were differentiated as Th1 or Th2 immunological type by the level of the TNF-α or IL-4 cytokines present in the patients’ serum, determined by indirect Enzyme-Linked Immunosorbent Assay (ELISA) kit (BD, Biosciences-Pharmingen, San Diego, CA, USA) and measured by an automatic microplate reader, V-Max (Molecular Devices, Sunnyvale, USA). When patients have levels twofold or higher of one cytokine compared to the other, they were considered serum Th1 or Th2 cytokine profile.

### Statistical analysis

Statistical analysis was performed using the GraphPad PRISM 5.04 program (La Jolla, CA, USA). The data relating to haematological parameters and the proportion of T cells and immunophenotyping of infected patients compared to non-infected controls were analysed by Mann–Whitney t test. Results were expressed as mean ± SEM (mean standard error). Values of *p *< 0.05 were considered statistically significant. For the analysis of correlation between parasitaemia and platelet count with different sub-populations of lymphocytes, the Spearman correlation coefficient test 95% CI, was performed.

## Results

### Haematological, biochemical and parasitological profile of patients infected with *Plasmodium vivax*

The data demonstrated that all patients with *P. vivax* infection evaluated in this study had some degree of thrombocytopaenia; some patients presented varying degrees of anaemia and some biochemical alterations (Table 1). All patients in this study reported more than one episode of malaria in their lifetime (n = 50). The data demonstrated that no patient had a severe malaria profile, according to the protocol established by the WHO.

**Table 1:** Haematological, biochemical and parasitological parameters of patients infected by *Plasmodium vivax*

Gender	Age (years)	RBCs (10^6^/mm^3^)	Hb g/dL	HCT %	Plat. mm^3^	Leuc. %	Lynf. %	Paras. mm^3^	GPT UI/L	GOT UI/L	Urea mg/dL	Cr mg/dL	BT mg/dL	TNF-α pg/mL	IL-4 pg/mL
M	29	4.35	13	38.5	66	6	34	305	47	26	22	0.78	0.4	9.4	110.4
M	18	4.46	14	40.1	96	7.5	14.7	235	82	50	22	0.65	0.9	9.2	127.3
F	19	4.09	11.1	33.4	109	4.7	18.4	1007	13	32	17	0.39	1.1	7.4	65.2
F	19	3.91	11.3	33.6	174	6.7	20.8	1012	13	27	20	0.4	0.62	10.5	62.9
F	22	3.63	11.4	34.2	82	8.1	24.9	505	25	24	24	0.62	0.43	60.8	24.3
M	38	4.81	14.7	43	122	6.1	32.2	1092	58	33	31	0.59	0.89	70.6	24.0
M	21	5.16	14.9	43.5	85	4.7	20.5	508	36	30	36	0.68	1.29	16.2	127.3
F	49	5.37	15.4	45.9	180	5.4	20.4	220	51	44	22	0.49	0.62	9.0	157.8
F	25	5.01	13.8	39.9	118	9.7	32.2	328	15	31	24	0.56	1.28	7.4	34.7
F	25	4.03	13.9	43	117	6.8	33	1020	12	23	23	0.34	0.7	7.1	88.9
M	19	4.25	11.3	34.7	129	7.3	23	513	11	17	17	0.43	0.57	10.6	96.8
M	36	5.36	15.4	44.4	84	9.5	16.3	355	68	44	24	0.53	0.97	11.4	235.7
F	50	4.68	14.2	41.8	73	6.9	10.5	1663	55	50	31	0.55	0.58	12.6	164.6
F	47	4.6	13.4	40.2	119	4.6	14.7	186	57	38	33	0.9	1.23	90.4	18.4
M	47	4.82	13.5	42.4	62	6.2	12.8	195	16	32	25	0.62	0.88	12.1	82.1
M	44	3.8	11.4	34.5	128	11.2	12.6	518	72	75	21	0.68	0.75	8.1	95.7
M	34	4.59	12.9	38.8	124	6	15.1	300	47	34	33	0.98	1.2	8.5	72.0
M	37	4.82	14.7	42.2	177	8.4	11.3	520	29	31	36	0.7	3.94	8.3	20.0
F	35	4.69	13.4	40	151	9.3	10.5	200	17	22	26	0.89	1.86	9.7	97.9
F	23	4.88	15.3	44.6	78	7.1	10.3	451	21	32	30	0.86	0.3	99.0	21.2
M	24	4.63	13.8	40.6	83	6	16.8	530	21	22	29	0.94	0.9	5.1	18.9
F	24	4.26	11.2	34.9	85	4.6	27.8	8380	35	33	27	0.67	1.16	14.4	62.9
F	34	3.7	10.3	32.3	128	5.6	19.7	270	12	19	15	0.45	0.4	9.0	65.2
M	20	5.29	16.3	48.5	90	8.5	14.7	1119	20	30	51	0.98	0.68	81.1	20.0
M	27	4.56	13.7	38.9	124	6.7	24.9	400	21	31	39	1.12	1.46	97.9	10.5
F	19	4.9	14	40.5	146	11.6	7.2	508	14	21	38	0.98	0.8	7.8	21.2
M	83	3.99	11.5	35.2	88	5	23	560	22	18	33	0.8	0.5	5.8	18.9
F	29	4.09	10.9	32.8	128	7.1	35.1	270	9	21	23	1.1	0.4	13.5	62.9
F	45	4.4	12	36.5	219	13.2	28.3	285	20	26	25	0.55	0.61	12.1	65.2
F	18	4.14	10	31.2	139	4.8	35.3	300	35	40	17	0.4	1.26	94.0	17.8
F	67	4.73	13.7	41	144	8.1	19	235	43	35	30	0.9	0.65	54.1	16.6
F	32	4.33	13.6	39.4	101	4.6	28.7	420	27	31	19	0.72	0.46	8.7	17.8
M	41	3.77	11.4	33.6	106	7.9	17.4	582	33	55	24	0.55	0.69	11.4	16.6
M	18	4.41	12.7	38.4	109	11.1	13	2304	38	29	46	0.8	0.7	7.4	66.3
F	23	5.11	13.3	41.6	77	5.9	12.1	1157	15	24	30	0.9	1.2	6.5	127.3
M	23	4.6	11	33	102	10.8	42	415	22	25	28	0.85	1.1	7.6	23.4
F	28	4.87	10.8	34.9	89	4.7	44	301	16	23	17	0.8	1.09	7.2	12.1
M	25	5.8	12.2	42	79	10	39.5	335	53	47	20	0.72	1.13	10.5	39.2
M	31	5.2	11.5	39.9	146	5.8	27	300	14	32	27	0.87	1.92	53.0	14.4
M	21	3.45	11.7	41.2	98	7.2	31.8	899	12	28	22	0.79	1.12	64.1	24.2
F	32	4.4	10.4	40	114	11.5	33.4	340	6	33	19	0.7	1.8	8.6	55.0
M	43	4.9	13.1	37.8	65	13	29	590	24	26	32	0.85	1.34	6.4	32.5
F	23	5.9	12.9	34.3	176	9.5	29.9	1010	30	28	24	0.6	0.7	6.8	11.0
F	19	5.7	13.9	44	53	4	15.2	300	15	40	27	0.5	0.5	8.4	92.3
M	25	5.12	14.6	43.6	78	11.4	12	460	12	24	33	0.9	1.4	12.9	58.4
M	29	4.25	12.6	37.9	47	2.7	21.5	654	28	24	31	1.0	1.48	16.3	43.7
F	37	4.09	13	38	70	3.9	18.3	623	13	30	18	0.87	0.9	26.6	43.7
M	44	4.46	12.2	38.3	98	4.3	22	465	18	32	19	0.8	0.85	6.1	35.8
F	43	5.14	14.3	44.1	90	4.5	30.3	355	20	26	21	0.74	1.23	10.4	64.3
F	59	5.03	14.8	45.4	110	4.4	32	420	18	27	27	0.5	0.6	8.2	80.1

Regarding the parasitaemia, 39 individuals presented mild parasitaemia (< 750 parasites/mm^3^), and 11 patients had high parasitaemia (> 751 parasites/mm^3^) (Table 1).

The age group of patients infected with *P. vivax* included 27 patients between ages 18 and 30 (54%) and 23 patients between ages 31 and 83 (46%). The number of infected men and women were virtually identical (n = 24 and 26, respectively).

### Comparative analysis of the number of platelets between groups and correlation of number of platelets and parasitaemia in patients infected with *Plasmodium vivax*

**Fig. 1:**
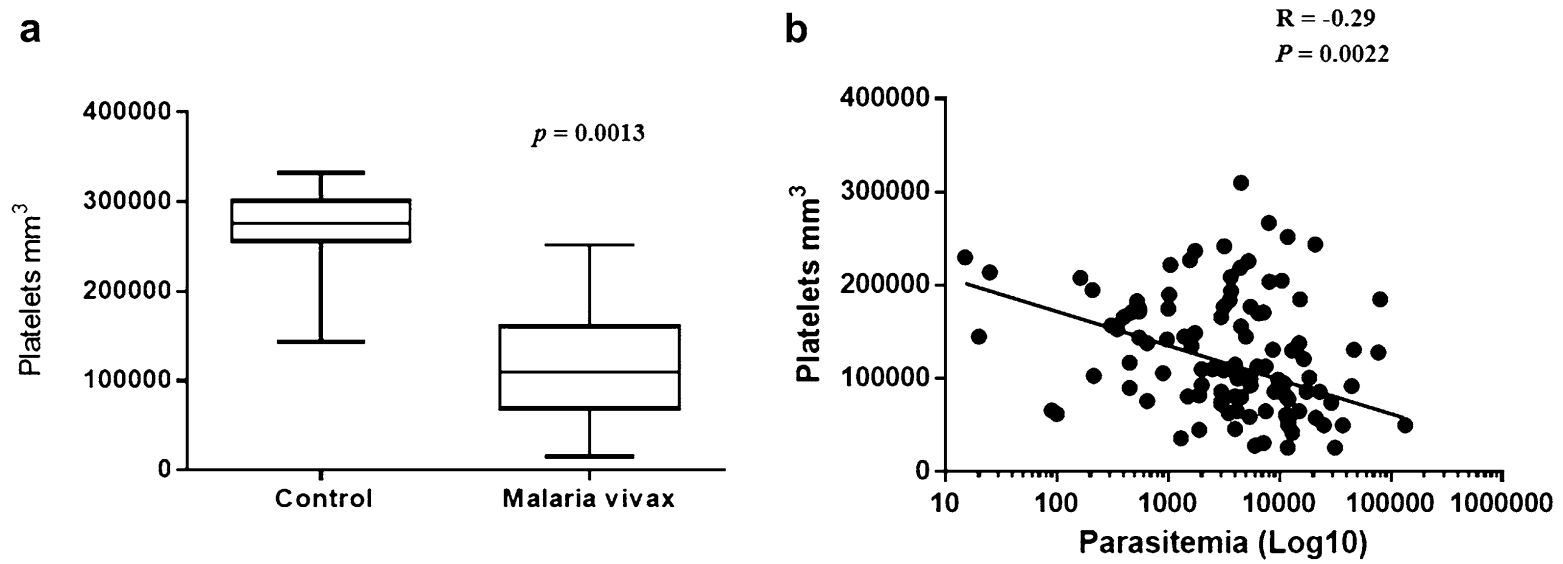
Comparison analysis of platelet counts among groups and correlation between platelet counts and parasitaemia in patients with malaria caused by *Plasmodium Vivax*. **a** Number of platelets (mm^3^) in patients infected with *P. vivax* (n = 50) and non-infected controls (n = 20). Mann–Whitney t test. **b** Presence of negative correlation between parasitaemia (mm^3^) and platelet counts in patients infected with *P. vivax*. Spearman correlation coefficient test

Patients with *P. vivax* infection presented thrombocytopaenia when compared to non-infected controls (*p *= 0.0013; Fig. 1a). Based on this result, to verify the correlation between the parasitaemia of patients with malaria caused by *P. vivax* and the number of platelets found in the peripheral blood at the time of diagnosis, the Spearman correlation coefficient test was performed. The data obtained indicated a negative correlation between the parasitaemia and the number of platelets of patients infected by *P. vivax* (Fig. 1b).

### Comparison of the cell profile of patients infected with *Plasmodium vivax* and non-infected controls T cells subpopulations CD3^+^CD4^+^IFN-γ^+^TNF-α^+^, CD3^+^CD4^+^IL-4^+^, CD3^+^CD4^+^IL-17^+^, CD4^+^CD25^+^CD127^+^FOXP3^+^ and CD4^+^CD25^+^CD127^−^FOXP3^+^

**Fig. 2:**
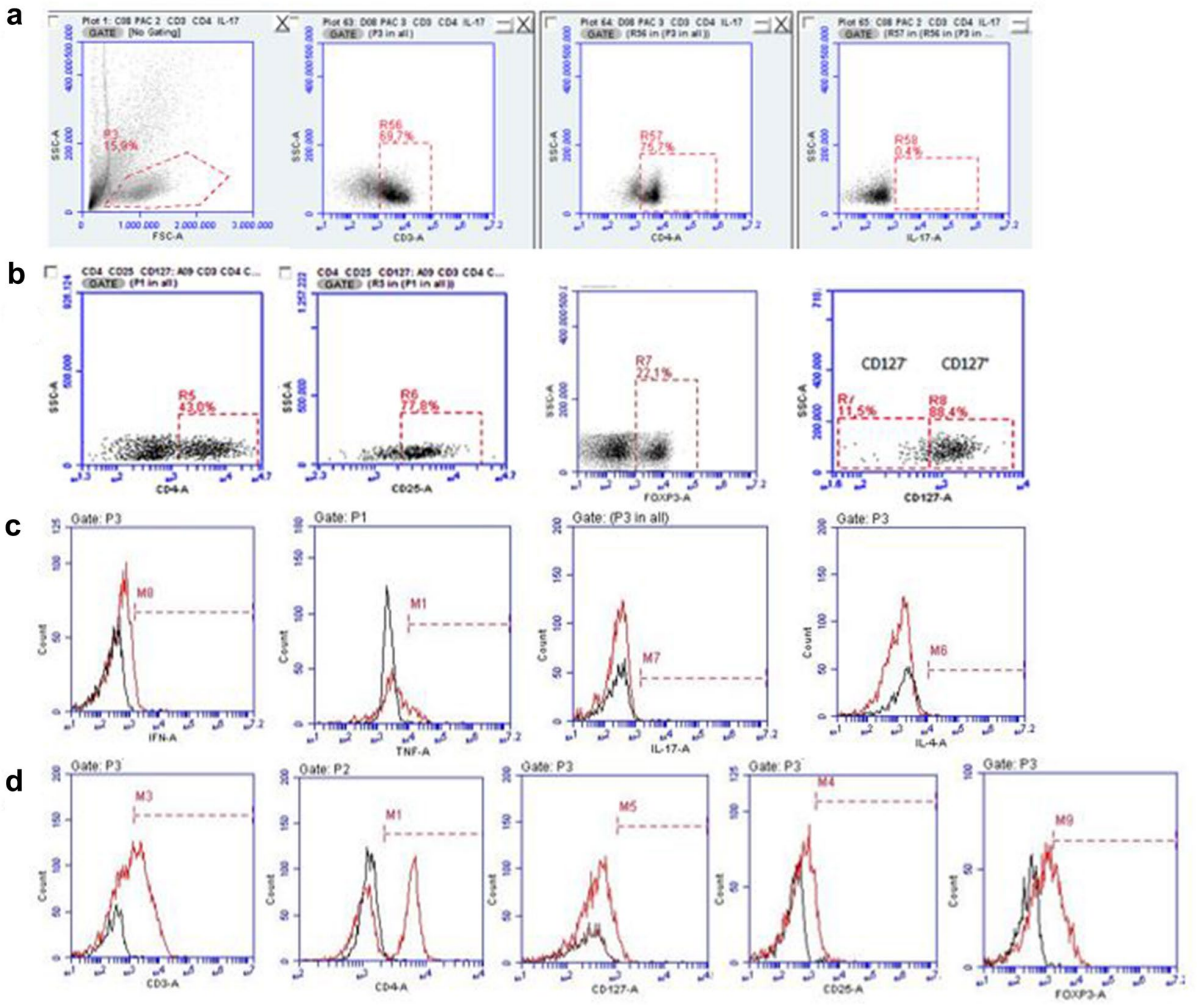
CD3^+^CD4^+^IL-17^+^, CD4^+^CD25^+^CD127^+^FOXP3^+^ and CD4^+^CD25^+^CD127^−^FOXP3^+^ cells analysis model used in flow cytometry. **a** Representative scheme of the analysis used to identify CD3^+^CD4^+^IL-17^+^ T cell sub-population through the identification of its cytokine and dot plots illustrate isotype control used to make the quadrants. **b** Representative scheme of the analysis used to identify Treg cell (CD4^+^CD25^+^CD127^+^FOXP3^+^) and activated Treg cells (CD4^+^CD25^+^CD127^−^FOXP3^+^). **c**, **d** Flow cytometry histograms show the isotype controls and cells with positive reactivity with human monoclonal antibodies. Black line shows the isotype control and red line shows cells

The leukogram of patients with malaria were evaluated and compared to the ones of healthy individuals and no significant statistical differences were noticed (Table 1). However, to evaluate the possible alterations in the different lymphocyte populations, the Th1, Th2, Th17, Treg, and activated Treg cells were investigated in the peripheral blood of the two groups, by flow cytometry as in the scheme below (Fig. 2).

**Fig. 3:**
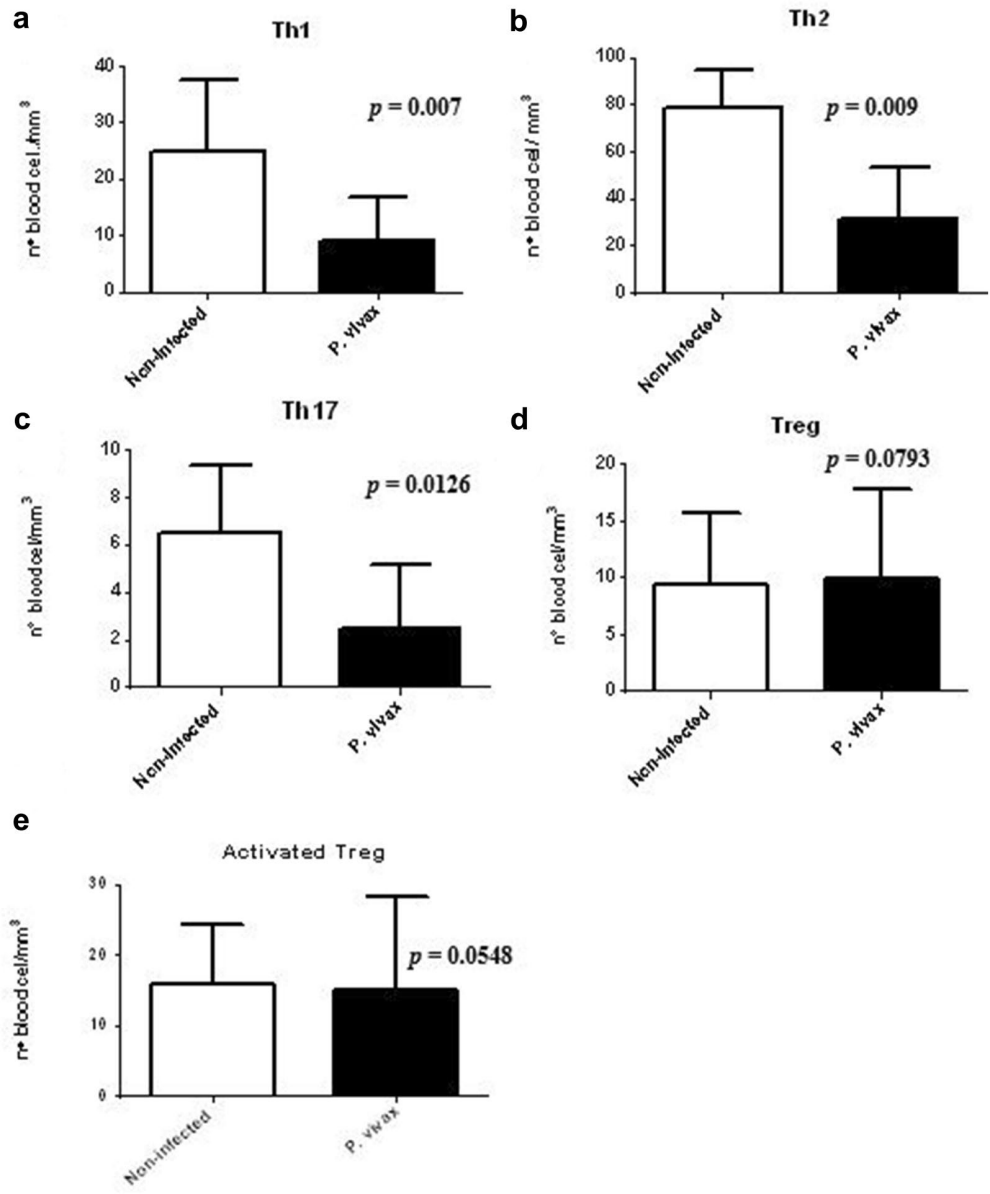
Analysis of the Th1, Th2, Th17, Treg and activated Treg cells profile. **a** Number of cells IFN-γ^+^/TNF-α^+^, **b** IL-4^+^, **c** IL-17^+^, **d**, **e** Tregs respectively, in the T cell sub-populations: Th1, Th2, Th17, Treg and activated Treg cells of patients infected with *P. vivax* (n = 50) and non-infected controls (n = 20). Mann–Whitney t test

The data obtained indicate that malaria patients presented a significant reduction in the cellular sub-populations of Th1 (*p *= 0.007), Th2 (*p *= 0.009) and Th17 (*p *= 0.0126, Fig. 3a–c). No statistical significance was demonstrated when comparing the number of Treg cell and activated Treg cells sub-population between groups of infected patients and noninfected controls (*p *> 0.05; Fig. 3d, e).

### Correlation between parasitaemia and T cell sub-populations: CD3^+^CD4^+^IFN-γ^+^TNF-α^+^, CD3^+^CD4^+^IL-4^+^, CD3^+^CD4^+^IL-17^+^, CD4^+^CD25^+^CD127^+^FOXP3^+^ and CD4^+^CD25^+^CD127^−^FOXP3^+^ in patients infected with *Plasmodium vivax*

The Spearman correlation coefficient test was performed to verify the presence of correlation between parasitaemia and T lymphocyte sub-populations identified in patients infected with *P. vivax*. However, the results did not present a statistically significant correlation in any subpopulation of T lymphocytes analysed (*p *> 0.05).

Next, to investigate the profile of the infectious process triggered by *P. vivax* in patients diagnosed with malaria, patients were divided into two groups using serum TNF-α and IL-4 levels to focus on the serum cytokine profile. It is known that high levels of serum TNF-α and IL-4 have correlated with Th1 and Th2 cytokine profile, respectively. Therefore, these two groups were nominated serum Th1 and Th2 cytokine profile, respectively. The same T cell sub-populations described above were analysed in each serum cytokine profile.

**Fig. 4:**
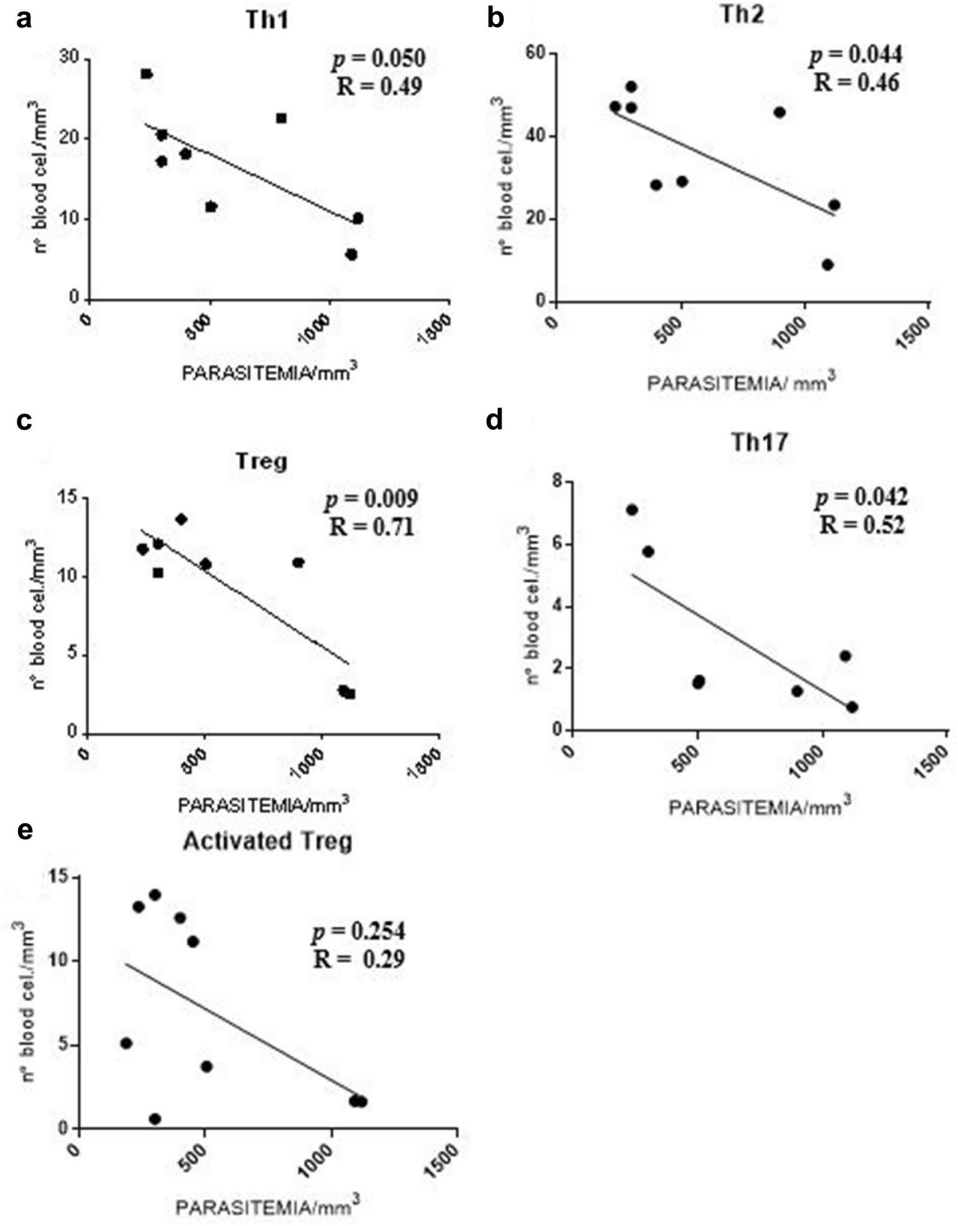
Correlation analysis between parasitaemia and T cell sub-populations in patients with serum Th1 cytokine profile. **a**–**d** Presence of negative correlation between parasitaemia (mm^3^) and T cell sub-populations Th1, Th2, Treg and Th17. **e** Absence of correlation between parasitaemia (mm^3^) and number of cells corresponding to the activated Treg cells subpopulation (n = 10). Spearman correlation coefficient test

Initially, evaluating patients with serum Th1 cytokine profile, the data indicated a negative correlation between the level of parasitaemia with the following T cell sub-populations: Th1 (*p *= 0.05), Th2 (*p *= 0.044), Treg (*p *= 0.009), and Th17 (*p *= 0.042, Fig. 4a–d, respectively). Only the activated Treg cells sub-population did not present a statistical significance with the level of parasitaemia (*p *> 0.05; Fig. 4e).

**Fig. 5:**
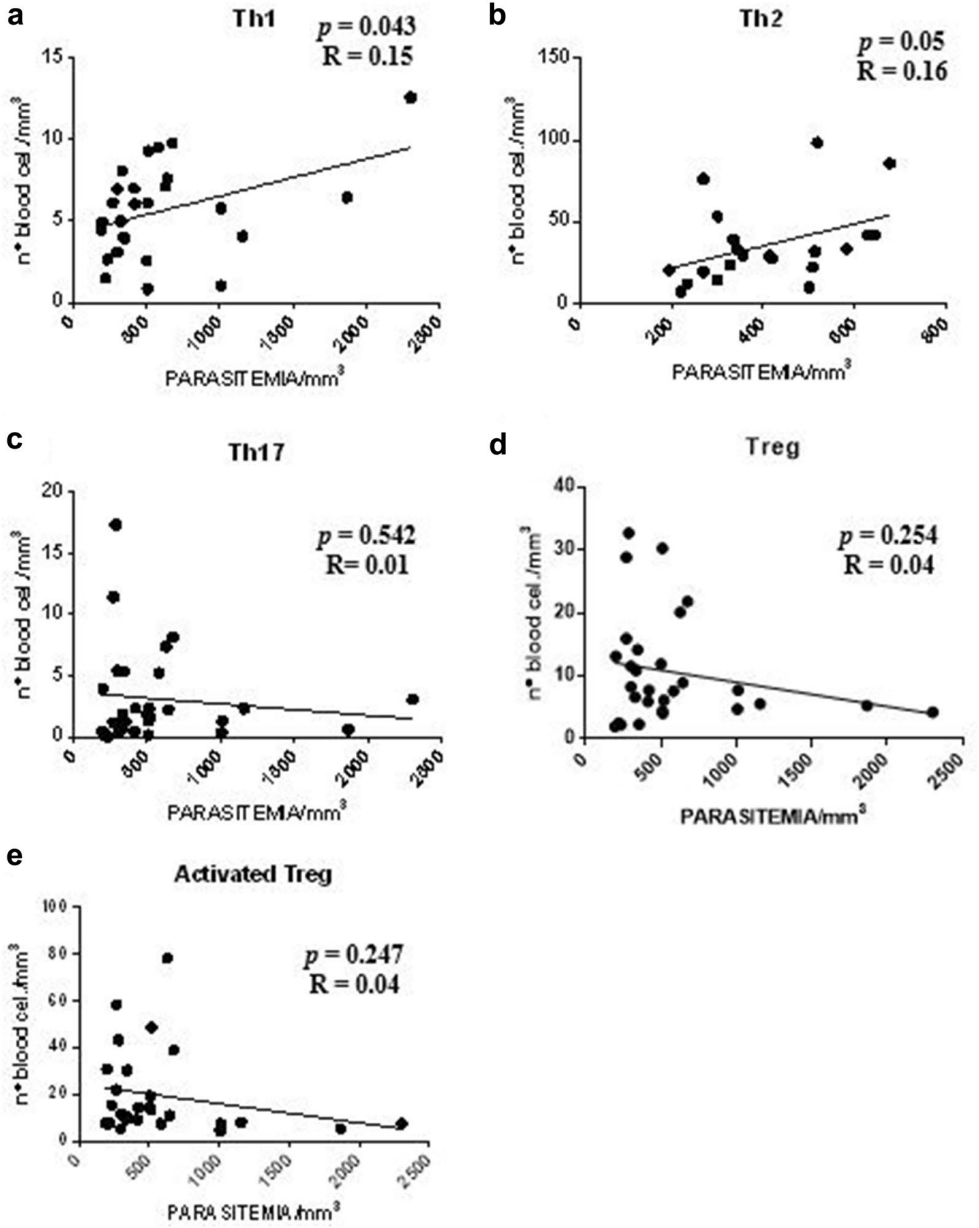
Correlation analysis between parasitaemia and T lymphocyte sub-populations in patients with serum Th2 cytokine profile. **a**, **b** Presence of positive correlation between parasitaemia (mm^3^) and sub-populations of Th1 and Th2 cells. **c**–**e** Absence of correlation between the level of parasitaemia (mm^3^) and the number of cells corresponding to the sub-populations of Th17, Treg and activated Treg cells (n = 40). Spearman correlation coefficient test

It was verified that the data obtained indicated the presence of a positive correlation between the level of parasitaemia and the sub-populations of Th1 and Th2 cells (*p *= 0.043 and *p *= 0.05; Fig. 5a, b, respectively) in patients with serum Th2 cytokine profile. No statistically significant difference was observed within the sub-populations of Th17, Treg and activated Treg cells (*p *> 0.05; Fig. 5c–e respectively).

### Correlation between platelets and T cell sub-populations: CD3^+^CD4^+^IFN-γ^+^TNF-α^+^, CD3^+^CD4^+^IL-4^+^, CD3^+^CD4^+^IL-17^+^, CD4^+^CD25^+^CD127^+^FOXP3^+^ and CD4^+^CD25^+^CD127^−^FOXP3^+^ in patients infected with *Plasmodium vivax*

The Spearman correlation coefficient test was performed to verify the presence of a correlation between the number of platelets and the T lymphocyte sub-populations, identified in patients infected with *P. vivax*. Therefore, no statistically significant correlation was found in patients infected with *P. vivax* (*p *> 0.05) when evaluating all of the T cell sub-populations described above.

**Fig. 6:**
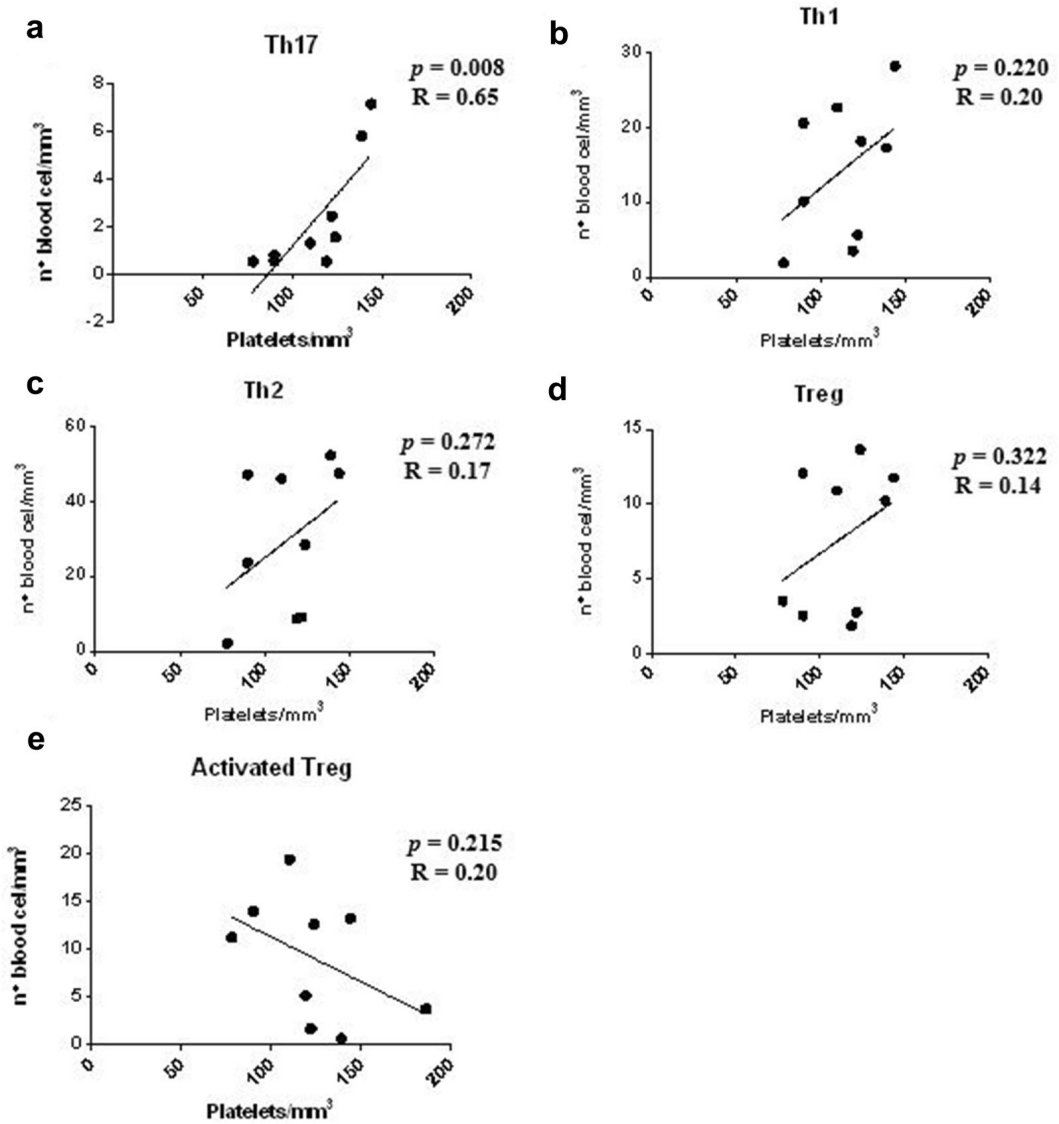
Correlation analysis between platelet count and T lymphocyte subpopulations in patients with serum Th1 cytokine profile. **a** Presence of positive correlation between the number of platelets (mm^3^) and the number of cells corresponding to the Th17 cell sub-population. **b**–**e** Absence of correlation between the number of platelets (mm^3^) and the number of cells corresponding to the T cell sub-populations: Th1, Th2, and Treg and activated Treg cells (n = 10). Spearman correlation coefficient test

However, when patients with serum Th1 cytokine profile were evaluated separately, the data obtained indicated a positive correlation between the number of platelets and the Th17 sub-population (*p *= 0.008; Fig. 6a). Furthermore, the sub-populations of T cells: Th1, Th2, Treg, and activated Treg cells (Fig. 6b–e, respectively) showed no correlation with the platelet counts of patients infected with *P. vivax*. (*p *> 0.05).

**Fig. 7:**
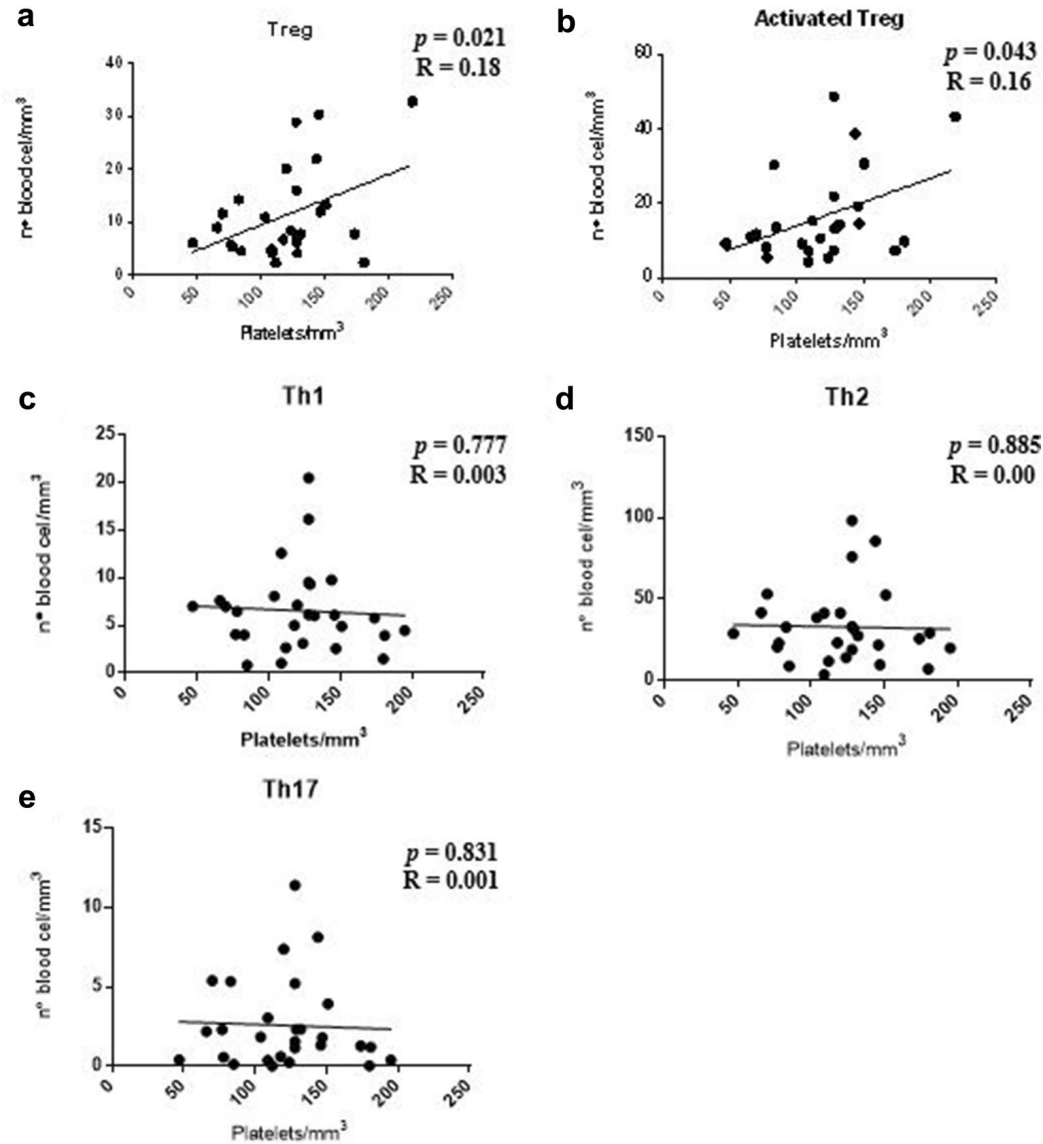
Correlation analysis between platelet counts and T lymphocyte sub-populations in patients with serum Th2 cytokine profile. **a**, **b** Presence of positive correlation between the number of platelets (mm^3^) and sub-populations of Treg cell and activated Treg cells. **c**–**e** Absence of correlation between the number of platelets (mm^3^) and the number of cells corresponding to the T cell sub-populations: Th1, Th2 and Th17 (n = 40). Spearman correlation coefficient test

The data obtained when patients with serum Th2 cytokine profile were evaluated showed a positive correlation between the number of platelets and Treg sub-populations and activated Treg cells (*p *= 0.021 and *p *= 0.043; Fig. 7a, b, respectively). No statistically significant correlation was found between the number of platelets and the T cell sub-populations: Th1, Th2 and Th17 (*p *> 0.05; Fig. 7c–e, respectively).

## Discussion

The infectious process of malaria is characterized by a complex interaction between the host’s immune response and parasite survival strategy. This study contributes to the delimitation of haematological, parasitological and immunological aspects of individuals infected with *P. vivax* who live in a region of Brazil where malaria is actively transmitted. When analysed as a whole, patients showed a variation in T lymphocyte sub-populations. However, the stratification of serum Th1 and Th2 cytokine profile strengthens the correlation analysis of haematology, parasitology and immunology in patients.

In the pathogenic process of malaria, the cellular immune response is mediated by Th1 profile T cell lymphocytes with IFN-γ production and Th2 profile, with IL-4 and IL-5 cytokines, to eliminate the parasite [26]. In this study, the leukogram of patients with *P. vivax* infection was analysed and the total number of leukocytes was within the normal range. These data were in agreement with Grynberg and collaborators [27]. However, when the sub-populations of CD4^+^ T lymphocytes Th1, Th2, Th17 and Treg were evaluated, the data showed that the numbers of these cells in patients infected by *P. vivax* were significantly lower in relation to the control group. Similar results were observed by Gonçalves and collaborators [28], including Hojo-Souza and collaborators [29]. The literature describes that the decrease in peripheral blood lymphocytes is usually observed in patients with acute malaria caused by *P. falciparum* and *P. vivax*, and that may be induced by lymph nodes sequestration and also death by apoptosis [23, 30].

The data showed a predominance of low parasitaemia for most patients. A study conducted in Porto Velho, Brazil, was in agreement with these results [31]. The literature indicates that the parasitaemia of the infected patients in endemic regions tends to be low or moderate, which directly relates to the severity of the clinical manifestations. In regions where transmission is low or unstable, immunity is also low and individuals may present severe disease [32].

In this study, patients with serum Th1 cytokine profile were observed with parasitaemia inversely proportional to the increased number of Th1, Th2, Th17, and Treg cell. The information gathered suggests that these T cell sub-populations suffer a clonal deletion during this infectious process when the parasitaemia is elevated. In agreement with these results, a study with individuals infected with *P. vivax* and low parasitaemia, show evidence of high serum levels of IFN-γ [33]. In malaria, IFN-γ has direct antiparasitic action and may act in synergism with TNF-α. The action of IFN-γ, as well as other pro-inflammatory cytokines, should be controlled and able to eliminate infection without causing damage to the host [34].

In the initial phase of infection, CD4^+^ T lymphocytes participate in the reduction of parasite density. During the course of infection, the proportion of Th2 cells increases, favouring the development of antibody-mediated immunity and, consequently, the reduction of parasitaemia and the resolution of the patent infection [35, 36]. This study demonstrates that the number of Th1 and Th2 cells was directly proportional to the parasitaemia during the infection by *P. vivax* in patients with serum Th2 cytokine profile, extending the view that these immune cells may contribute to direct immunological anti-parasitic mechanisms.

The data established in this study, associated with the literature may indicate that in patients with a serum Th1 cytokine profile, activated Treg cells would be newly migrated cells that would not have a correlation with parasitaemia. The sub-population of Treg cells are possibly memory cells, which have an inverse correlation with parasitaemia. Studies indicate that by using CD127, the newly activated effector cells and the memory cells of the Treg population can be more clearly distinguished, since only newly activated Treg have a low expression of CD127. The memory cells have high expression of this marker and traditional effector cells rapidly re-express this marker after activation [15, 37]. These results are consistent with those recently published in a study with neonates of women infected with *P. falciparum*. In this situation, the frequency of both Treg cells (defined by the CD25^+^CD127^+^FOXP3^−^ and CD25^+^CD127^−^FOXP3^+^ phenotypes, respectively) did not range from the placental parasitaemia, and decreased significantly as the level of placental inflammation increased [38].

Treg cells failed to show statistically significant differences when analysed in patients with a serum Th2 cytokine profile. Perhaps these cells are not phenotypically dysregulated during *P. vivax* infection. Evidence indicates that Treg cells can suppress T cell responses by producing IL-10 and TGF-β. Also, Treg cells can suppress B cell maturation and differentiation directly or indirectly by regulating IL-2 or IL-4 production [39]. The absence of deregulation suggests that the potential of these cells is maintained and may contribute to infection control and clinical immunity to *P. falciparum* or *P. vivax* infection [40]. Another study conducted with DEREG mice analyses the effect of CD4^+^FOXP3^+^ Treg on the course of non-lethal *Plasmodium yoelii* infection in BALB/c mice. The study reveals that the depletion of Treg cells reduced the level of parasitaemia and increased the activation of CD4^+^ and CD8^+^ T cells, indicating that Treg cells play a crucial role in the control of immune responses to parasitic infection [41].

The findings in this study showed that patients infected by *P. vivax* presented a significant decrease of Th17 in relation to the control group. Another observation made, was the statistically significant decrease in the number of Th17 cells when correlated with parasitaemia in patients infected with *P. vivax* with serum Th1 cytokine profile. Those data do not provide any clear conclusion regarding the role of these cells in the protection or pathogenesis of this disease in relation to parasitaemia. Bueno and collaborators [42], in turn, showed that Th17 cells were detected in patients with infection by *P. vivax* in the state of Amazonas. These findings correlated with the high production of IFN-γ, IL-10 and TGF-β. The production of IL-17 by CD4^+^ T cells was previously described during malaria infection in Treg cells [17, 42]. A study demonstrated in vitro that there may be a relationship between Th17 and Treg, and the balance between these cells may lead to susceptibility or resistance against other models of inflammation [43].

When compared to the control group patients infected with *P. vivax* presented thrombocytopaenia and a negative correlation with the level of parasitaemia in the group of individuals infected with *P. vivax*. Similarly, studies present an increase in the number of platelets parallel to the reduction of parasitaemia in patients with malaria caused by *P. falciparum* [6] or by *P. vivax* [2].

A correlation analysis between the number of platelets and the specific lymphocyte subpopulations present in the infectious process of individuals with malaria caused by *P. vivax*, was performed to verify whether the activation of these sub-populations could contribute to thrombocytopaenia. Several studies have tried to clarify the origin of this vital haematological alteration. A concomitant increase in the number of Th17 and platelet cells in patients infected with *P. vivax* with serum Th1 cytokine profile was observed. The IL-17 induces the expression of various mediators of inflammation [19]. However, the inflammatory response to malaria results in erythrocyte modifications that may increase changes in blood flow, including loss of normal discoid shape, increased membrane stiffness and high permeability, facilitating the survival of the parasite within the host cell and increasing the virulence of the disease [44, 45]. One study demonstrated that symptomatic patients infected with *P. vivax* who were treated with IL-17 displayed a reduction in blood viscosity, indicating the possible use of this cytokine as a potential immunomodulatory agent [33].

Furthermore, the analysis performed in patients with serum Th2 cytokine profile, verified a significant positive correlation with the number of platelets and Treg cells and activated Treg cells in the patients infected with *P. vivax*. A study of children in Kenya, with *P. falciparum* infection, showed a significant and independent inverse correlation between platelet counts and plasma IL-10 [46]. It is speculated in this work that IL-10 could, by reducing the number of circulating platelets, prevent the adhesion of parasitized red blood cells to the vascular endothelium, as if thrombocytopaenia could represent a defense mechanism against severe malaria. In another study, thrombocytopaenia in patients with *P. vivax* infection was associated with increased IL-1, IL-6, IL-10, and TGF-β [47]. Thus, in addition to immunological profiles, other biological mechanisms may also be important for the regulation of thrombocytopaenia.

The epidemiological data and laboratory analysis of patients with *P. vivax* infection were verified. The literature reports more frequent cases of malaria caused by *P. vivax* in male individuals, however, it is correlated with economic activity, usually in *garimpo* and agriculture [48]. The area studied was an endemic urban area, therefore, there was no gender prevalence in the data obtained for this study. All patients in this study reported more than one malaria episode during their lifetime. However, no adjustments were made according to previous malaria experience, which may be a limitation for this study. With regard to the laboratory analysis, biochemical tests and blood count were performed to identify suggestive cases of severe malaria caused by *P. vivax*, using established standards for *P. falciparum* infection [49], as already done in other studies [50]. The most common complications in malaria are haematologic changes, which are involved in disease severity and fatality [51]. The biochemical and haematological data of the patients in this study did not reach severity parameters.

## Conclusions

The data rendered in this study are related to the aspects of the immune response and its activation against parasite load and platelet activation during malaria caused by *P. vivax.* Considering the different defence mechanisms of the host against an infection, it is possible to hypothesize that the lymphocytic profile is dependent on parasitaemia and the number of platelets for its differentiation and activation. Further studies will be important to elucidate in more detail the mechanisms of action triggered by the immune system against *P. vivax* infection.

## Additional file

**Additional file 1: Table S1.** Demonstration of monoclonal antibodies used in cellular immunophenotyping analyzes.
