# Supplementary material for: Analysis of the lymphocyte cell population during malaria caused by Plasmodium vivax and its correlation with parasitaemia and thrombocytopaenia
Source: Malar J. 2018 Aug 20;17:303. doi: 10.1186/s12936-018-2443-x (PMC6102853; doi:10.1186/s12936-018-2443-x)
Supplement: Supplementary file 1 — Additional file 1: Table S1. Demonstration of monoclonal antibodies used in cellular immunophenotyping analyzes. [file 12936_2018_2443_MOESM1_ESM.docx]

**Additional file 1: Table S1**

**Demonstration of monoclonal antibodies used in cellular immunophenotyping analyzes**

| **Monoclonal** | **Manufacturer** | **Lot number** | **Concentration** | **Target population** |
| --- | --- | --- | --- | --- |
| Mouse Anti-Human CD3 PerCP | BD-Pharmigen^TM^ | 552851 (clone SP34-2) | 20 μL | Lymphocytes T |
| Mouse Anti-Human CD4 APC | BD-Pharmigen^TM^ | 561841 (clone RPA-T4) | 5 μL | Lymphocytes CD4^+^ |
| Mouse Anti-Human CD4 PerCP-Cy ^TM^ 5.5 | BD-Pharmigen^TM^ | 552838 (clone L200) | 20 μL | Lymphocytes CD4^+^ |
| Mouse Anti-Human CD127 Alexa Fluor® 647 | BD-Pharmigen^TM^ | 558598 (clone HIL-7R-M21) | 20 μL | Lymphocytes CD4^+^, Treg |
| Mouse Anti-Human CD25 FITC | BD-Pharmigen^TM^ | 555431 (clone M-A251) | 20 μL | Lymphocytes CD4^+^, Treg |
| Mouse Anti-Human IFN- γ^+^ PE | BD-Pharmigen^TM^ | 559327 (clone B27) | 20 μL | T cells, mainly Th1 |
| Mouse Anti-Human IL-4 PE | BD-Pharmigen^TM^ | 340451 (clone 3010.211) | 20 μL | Mainly Th2 |
| Mouse Anti-Human IL-17 PE | BD-Pharmigen^TM^ | 560436 (clone SCPL1362) | 20 μL | Lymphocytes CD4^+^, Th17 |
| Mouse Anti-Human TNF FITC | BD-Pharmigen^TM^ | 552889 (clone MAb11) | 20 μL | T cells, mainly Th1 |
| Mouse Anti-Human FOXP3 PE | BD-Pharmigen^TM^ | 560852 (clone 236ª/E7) | 5 μL | Lymphocytes CD4^+^, Treg |
| Mouse IgG1 K Isotype  Control Alexa Fluor® 647 | Lifetechnologies | 557714 (clone MOPC-21) | 20 μL |  |
| Mouse IgG1 K Isotype  Control APC | BD-Pharmigen^TM^ | 555751 (clone MOPC-21) | 5 μL |  |
| Mouse IgG1 K Isotype  Control PE | BD-Pharmigen^TM^ | 559320 (clone MOPC-21) | 20 μL |  |
| Mouse IgG1 K Isotype  Control FITC | BD-Pharmigen^TM^ | 555748 (clone MOPC-21) | 20 μL |  |
| Mouse IgG1 K Isotype  Control PerCP | BD-Pharmigen^TM^ | 559425 (clone MOPC-21) | 20 μL |  |
| Mouse IgG1 K Isotype  Control PE | BD-Pharmigen^TM^ | 551436 (clone MOPC-21) | 20 μL |  |
| Mouse IgG1 K Isotype  Control FITC | BD-Pharmigen^TM^ | 556649 (clone MOPC-21) | 20 μL |  |
| Mouse IgG1 K Isotype  Control PerCP-Cy^TM^ 5.5 | BD-Pharmigen^TM^ | 552834 (clone MOPC-21) | 20 μL |  |

Source: From the researcher. *μL for each 1x10^6^ cells in 100 μL of volume.
